# Sleeve Gastrectomy-Induced AMPK Activation Attenuates Diabetic Cardiomyopathy by Maintaining Mitochondrial Homeostasis *via* NR4A1 Suppression in Rats

**DOI:** 10.3389/fphys.2022.837798

**Published:** 2022-03-10

**Authors:** Songhan Li, Shuohui Dong, Qian Xu, Bowen Shi, Linchuan Li, Wenjie Zhang, Jiankang Zhu, Yugang Cheng, Guangyong Zhang, Mingwei Zhong

**Affiliations:** ^1^Department of General Surgery, Shandong Qianfoshan Hospital, Cheeloo College of Medicine, Shandong University, Jinan, China; ^2^Department of General Surgery, The First Affiliated Hospital of Shandong First Medical University, Jinan, China

**Keywords:** sleeve gastrectomy, diabetic cardiomyopathy, mitochondrial homeostasis, oxidative stress, AMPK, NR4A1

## Abstract

Diabetic cardiomyopathy (DCM) is characterized by impaired diastolic and systolic myocardial performance and is a major cause of morbidity and mortality in patients with diabetes. Surgical bariatric procedures, such as sleeve gastrectomy (SG), result in remission of type 2 diabetes (T2DM) and have benefits with myocardial function. Maintaining cardiac mitochondrial homeostasis is a promising therapeutic strategy for DCM. However, whether SG surgery affects mitochondrial function and its underlying mechanism remains unclear. This study aimed to investigate the effect of SG surgery on mitochondrial homeostasis and intracellular oxidative stress in rats with DCM. We also examined cellular phenotypes and molecular mechanisms in high glucose and high fat-stimulated myocytes. The rat model of DCM was established by high-fat diet feeding and low-dose streptozotocin injection. We observed a remarkably metabolic benefit of SG, including a reduced body weight, food intake, blood glucose levels, and restored glucose tolerance and insulin sensitivity post-operatively. Also, SG ameliorated the pathological cardiac hypertrophy, myocardial fibrosis and the dysfunction of myocardial contraction and diastole, consequently delayed the progression of DCM. Also, SG restored the mitochondrial dysfunction and fragmentation through the AMPK signaling activation mediated nuclear receptor subfamily 4 group A member 1 (NR4A1)/DRP1 suppression *in vivo*. H9c2 cardiomyocytes showed that activation of AMPK could reverse the mitochondrial dysfunction somehow. Collectively, our study provided evidence that SG surgery could alleviate mitochondrial dysfunction in DCM. Moreover, AMPK-activated NR4A1/DRP1 repression might act as a significant reason for maintaining mitochondrial homeostasis in the myocardium, thus contributing to morphological and functional alleviation of DCM.

## Introduction

Diabetic cardiomyopathy (DCM) is defined as a distinct disease entity that occurs in patients with diabetes mellitus (DM) independent of coronary artery disease, hypertension, or valvular heart disease (Jia et al., 2018; Tan et al., 2020). During the early phases of diabetes, an absence of insulin or insulin resistance initiates a metabolic alteration in cardiomyocytes, which leads to the activation of fatty acid uptake and β-oxidation to ensure adequate adenosine-triphosphate (ATP) generation. However, as the disease progresses, β-oxidation cannot sufficiently utilize all available fatty acids, resulting in intracellular lipid collection and lipotoxicity. Increased fatty acid concentrations subsequently result in mitochondrial dysfunction and an elevated accumulation of reactive oxygen species (ROS) (Tan et al., 2020). Ultimately, these pathological transformations result in cardiomyocyte death, inflammation, and fibrotic remodeling (Jia et al., 2018). Adenosine monophosphate-activated protein kinase (AMPK) plays an important role in metabolic diseases (Hoffman et al., 2015). Under DCM conditions, the activation of energy deprivation sensors, sirtuin-1 (SIRT1) and phosphorylation of AMPK can promote the removal of dysfunctional mitochondria and peroxisomes to reverse the development of cardiomyopathy (Packer, 2020a). Thus, AMPK signaling shows promising potential as a novel target for DCM treatment.

At present, bariatric surgery, such as sleeve gastrectomy (SG), is one of the most prevalent and valid therapies for obesity and related metabolic disorders (Ulrich-Lai and Ryan, 2014). Ample evidence has shown that SG leads to efficient remission of type 2 diabetes mellitus (T2DM) and its related complications (Mingrone et al., 2015; Rubino et al., 2016). Several studies have revealed that SG has benefits in terms of systolic and diastolic myocardial function in patients with DCM and in rodent models (Leung et al., 2016; Huang et al., 2018). In addition, bariatric surgery can facilitate Ca^2+^ homeostasis and attenuate myocardial autophagy, restore myocardial glucose uptake with myocardial glucose transporter four translocation, and activate MAPK signaling pathway to alleviate DCM (Huang et al., 2018, 2019; Xu et al., 2021). However, it remains unclear whether SG ameliorates DCM by maintaining mitochondrial homeostasis, and further supporting evidence is required.

To clarify these issues, we established a DCM rat model through high-fat-diet (HFD) administration and intraperitoneal injection of low-dose streptozotocin (STZ), with chow diet-fed rats serving as negative controls. SG or sham surgery was performed to investigate the effects of SG on mitochondrial structure and function in rats with DCM. Moreover, we determined that AMPK signaling-mediated nuclear receptor subfamily 4 group A member 1 (NR4A1) suppression might induce mitochondrial homeostasis to protect cardiomyocytes against diabetes-induced mitochondrial dysfunction and oxidative stress. Furthermore, the metabolic phenotypes and protective role of the AMPK pathway were examined in high glucose (HG) and palmitate (PA)-stimulated myocytes.

## Materials and Methods

### Reagents and Antibodies

D-Glucose (50-99-7) and STZ (S0130) were purchased from Sigma, Shanghai, China. We also purchased A-769662 (HY-50662) and 10-*N*-nonyl acridine orange (NAO; HY-D0993) from MedChemExpress, Shanghai, China. A hematoxylin and eosin staining kit (C0105S), an Oil Red O staining kit (C0158S), an ATP detection kit (S0026), a JC-1 staining kit (C2003S), and 2,7-dichlorodihydrofluorescein diacetate (DCFH-DA) probes (S0033S) were purchased from Beyotime, Shanghai, China. A diaminobezidin (DAB) staining kit (ZLI-9017) was purchased from ZSGB-BIO, China. A Mito-Tracker staining kit, Cambridge, United Kingdom (ab112145) was purchased from Abcam (Cambridge, United Kingdom). A total protein extraction kit for muscle (SA-06-MS) and a mitochondria isolation kit for muscle tissues/cultured muscle cells (MM-038) was purchased from Invent Biotechnologies, Beijing, China. A bicinchoninic acid (BCA) protein assay kit (KGP902) was purchased from KeyGEN, Jiangsu, China. TRIzol reagent (15596018) was purchased from Invitrogen, Carlsbad, CA, United States. A ReverTra Ace qPCR RT kit (FSQ-101) and SYBR Green Real-time PCR Master Mix (QPK-201) were purchased from Toyobo, Japan. The following antibodies were used: collagen I (Affinity, AF7001), collagen III (Affinity, AF0136), NR4A1 (Proteintech, 25851-1-AP), DRP1 (Affinity, DF7037), nuclear factor erythroid-2-related factor 2 (NRF2; Proteintech, 16396-1-AP), heme oxygenase 1 (HO-1; Proteintech, 27282-1-AP), CPT-1α (Proteintech, 15184-1-AP), PPARα (Proteintech, 15540-1-AP), superoxide dismutase-2 (SOD2; Abcam, ab68155), ACC1 (Proteintech, 21923-1-AP), phosphate-ACC1 (CST, 11818), AMPKα (CST, 5832), phosphate-AMPK (CST, 50081), SIRT1 (Proteintech, 13161-1-AP), NDUFB8 (Abcam, ab192878), SDHB (Abcam, ab175225), UQCRC2 (Proteintech, 14742-1-AP), MTCO1 (Abcam, ab14705), ATP5A (Proteintech, 14676-1-AP), HSP60 (Proteintech, 15282-1-AP) and β-Tubulin (Proteintech, 10094-1-AP).

### Cells Culture

The H9c2 rat embryonic cardiomyocyte line was donated by the Key Laboratory of Cardiovascular Remolding and Function Research of Shandong University (Jinan, China) and was validated by STR profiling. H9c2 cells were cultured in HG Dulbecco’s modified Eagle’s medium (DMEM; Gibco, Shanghai, China) supplemented with 10% (v/v) fetal bovine serum (Gibco, Shanghai, China), 100 U/mL penicillin, and 100 mg/mL streptomycin (KeyGEN, Jiangsu, China) at 37°C in a 5% CO_2_ humidified incubator. The cells were passaged every 2–3 days using 0.25% trypsin-EDTA for dissociation. To study the effects of high glucose (HG) and high fat conditions on H9c2 cells *in vitro*, cells were starved for 12 h and treated with 33.3 mM D-glucose (Sigma, Shanghai, China) and 500 μM palmitate (PA, Sigma, Shanghai, China) for 48 h. Furthermore, to determine the role of the AMPK pathway in H9c2 cells cultured under HG and high fat conditions, 10 μM A-769662, an AMPK activator (MedChemExpress, Shanghai, China), was added for 48 h.

### Animals

Male Wistar rats at the age of about 6 weeks were purchased from Beijing Weitong Lihua Experimental Animal Technology (Beijing, China) and housed in the Animal Center of Shandong Qianfoshan Hospital affiliated to Shandong University. Rats were raised in an environment with access to food and water under 12-h light/dark cycle at a constant temperature of 22°C. After 1 week of acclimatization, rats were randomly separated into three groups; one group was fed with conventional feed (control), whereas the other two groups were fed an HFD (60% of calories as fat; Xietong, Nanjing, China) for 4 weeks to induce obesity and insulin resistance. To further establish the DCM rat model, we intraperitoneally injected rats with STZ (Sigma, Shanghai, China) dissolved in sodium citrate buffer (pH 4.5; Solarbio, Beijing, China) at a dose of 35 mg/kg after 12 h fasting. One week after STZ injection, fasting blood glucose (FBG) level exceeding 11.1 mmol/L was considered to be indicative of successful modeling. All procedures were approved by the Institutional Animal Care and Use Committee of Shandong Qianfoshan Hospital affiliated to Shandong University. All animal studies complied with relevant ethical regulations for animal testing and research.

### Surgical Procedures

The established T2DM rat models were subjected to SG or sham surgery randomly. Briefly, rats were fed a liquid diet with 10% Ensure (Abbott Laboratories, Abbott Park, IL, United States) for 48 h, and they underwent fasting for 12 h before surgery. After administration of anesthesia with isoflurane (3% isoflurane for induction and 2% isoflurane for maintenance), SG and sham surgery were performed according to the standard process (Cheng et al., 2019), SG was started with a 4-cm midline abdominal incision at the xiphoid process, after ligation and transection of the vessels of the greater curvature, the greater curvature of the stomach from the cardia to the pylorus was separated. Entire glandular stomach and most of the gastric body was excised, leaving approximately 30% volume of the total stomach. Residual stomach was interruptedly sutured with 7–0 silk and abdominal was closed with 5–0 silk suture after careful examination. As for sham surgery, the abdomen was incised for the exposure of the stomach, esophagus, and small intestine. No other procedure was performed and operative time was prolonged to induce a comparable degree of anesthetic stress to the operated rats. The sham surgery group was used to eliminate the effects of surgical stress and anesthesia. After surgery, the rats were put into separate cages, and the diet was gradually transitioned from a liquid diet to a chow diet. Vital signs were monitored, and surgery-related complications were closely detected. Food intake and body weight were recorded every week. All surgical procedures complied with relevant ethical regulations for animal testing and research.

### Oral Glucose Tolerance Test, Insulin Tolerance Test, and Homeostasis Model of Assessment for Insulin Resistance Index

To assess insulin sensitivity, an oral glucose tolerance test (OGTT) and insulin tolerance test (ITT) were performed and homeostasis model of assessment for insulin resistance index (HOMA-IR) was measured before surgery and 8 weeks after surgery. Blood glucose level from the rat tail vein was detected using a glucometer (ACCU-CHEK Performa; Roche, Shanghai, China) at baseline and 10, 30, 60, and 120 min after the administration of intragastric gavage of 20% glucose (1 g/kg) for the OGTT or intraperitoneal injection of human insulin (0.5 IU/kg) for the ITT. HOMA-IR was evaluated using the following formula: HOMA-IR = fasting plasma glucose (mmol/L) × fasting insulin (mIU/L)/22.5.

### Assessment of Cardiac Function

To assess the cardiac function of the rats, transthoracic echocardiography imaging was performed on 2% isoflurane-anesthetized rats using a VEVO 3100 ultrasound machine (VisualSonics, Toronto, ON Canada). The main parameters included left ventricular end-diastolic diameter (LVEDd), left ventricular ejection fraction (LVEF), fractional shortening (FS), and early to late mitral diastolic flow ratio (E/A).

### Histology and Immunohistochemistry

After the rats were sacrificed, fresh myocardial tissue samples were fixed in formalin to prepare paraffin-embedded blocks, which were cut into 5-μm paraffin sections for histological analysis. The paraffin sections were stained with hematoxylin and eosin (H&E), Masson’s trichrome, Sirius red, and wheat germ agglutinin (WGA) to assess the standard histology, distribution of collagen, extent of fibrosis, and morphology of the cardiomyocyte membrane, respectively. For IHC, the paraffin sections were sequentially subjected to dewaxing, dehydration with gradient alcohol, microwave thermal repair in citrate buffer (0.01 M, pH 6.0), endogenous peroxidase blocking, and blocking in 10% goat serum in phosphate-buffered saline. The sections were then incubated with primary antibodies, anti-collagen I (Affinity, 1:1,000) or collagen III (Affinity, 1:500), overnight at 4°C. The next day, sections were incubated with HRP-conjugated anti-rabbit IgG (ZSGB-BIO, Beijing, China), and reactions were detected by DAB staining (ZSGB-BIO, Beijing, China). The nuclei were stained with hematoxylin and differentiated in 1% acid alcohol, after which the sections were rinsed with running water. Finally, the slides were sealed with a neutral gel. Images were acquired using an Axio Scope A1 microscope (Zeiss, Oberkochen, Germany).

### Mitochondrial Visualization by Transmission Electron Microscopy and MitoTracker

One-millimeter cubes of left ventricular myocardial tissue were pre-fixed in 2.5% glutaraldehyde overnight at 4°C and post-fixed in 1% osmium tetroxide for 2 h at 4°C. Fixed samples were subjected to dehydration, embedding, polymerization, and ultrathin section preparation, followed by staining with uranyl acetate and lead citrate. Finally, images were acquired using a Hitachi HT-7800 transmission electron microscope (Hitachi, Tokyo, Japan).

To visualize the mitochondria of the H9c2 cells after the HG and high fat conditions treatment, H9c2 cells were incubated with a solution of MitoTracker (Abcam, Cambridge, United Kingdom) for 1 h at 37°C when the cells reached 80% confluency. After incubation, images were acquired using an Axio Scope A1 microscope (Zeiss, Oberkochen, Germany).

### Protein Extraction and Western Blot Analysis

Heart tissue proteins were extracted using a total protein extraction kit for muscles (Invent Biotechnologies, Beijing, China). Isolation of mitochondria was prepared using a mitochondria isolation kit for muscle tissues/cultured muscle cells (Invent Biotechnologies, Beijing, China). Cell proteins were lysed in RIPA lysis buffer (Solarbio, Beijing, China) with a protease inhibitor (KeyGEN, Jiangsu, China) when confluency reached 80%. The lysates of the tissue samples or cells were then centrifuged at 12,000 × *g* for 20 min, and the supernatant was collected. Protein concentrations were determined using a BCA protein assay kit (KeyGEN, Jiangsu, China). Boiled protein solutions were separated by 10% SDS-PAGE (Bio-Rad, Hercules, United States) and transferred to a 0.45-μm PVDF membrane (Millipore, Cork, Ireland). After 1-h blocking in 5% skim milk powder (Beyotime, Shanghai, China), the membrane was incubated with the following primary antibodies at 4°C overnight: NR4A1 (Proteintech, 1:1,000), DRP1 (Affinity, 1:1,000), NRF2 (Proteintech, 1:2,000), HO-1 (Proteintech, 1:1,000), CPT-1α (Proteintech, 1:1,000), PPARα (Proteintech, 1:1,000), SOD2 (Abcam, 1:5,000), ACC1 (Proteintech, 1:1,000), phosphate-ACC1 (CST, 1:1,000), AMPK (CST, 1:1,000), phosphate-AMPK (CST, 1:500), SIRT1 (Proteintech, 1:1,000), NDUFB8 (Abcam, 1:5,000), SDHB (Abcam, 1:100,000), UQCRC2 (Proteintech, 1:1,000), MTCO1 (Abcam, 1:1,000), ATP5A (Proteintech, 1:1,000), HSP60 (Proteintech, 1:10,000) and β-Tubulin (Proteintech, 1:5,000). The secondary antibody was incubated for 1.5 h at room temperature. Proteins were detected by enhanced chemiluminescence (Millipore, Billerica, MA, United States) using an LI-COR Odyssey Imager (LI-COR Biosciences, Lincoln, United States).

### RNA Extraction and Reverse Transcription-Quantitative Polymerase Chain Reaction

Total RNA was extracted from heart tissues and H9c2 cells using TRIzol reagent (Invitrogen, Carlsbad, CA, United States), and RNA concentrations were measured using a NanoDrop spectrophotometer (NanoDrop Technologies, Wilmington, United States). cDNA synthesis was performed using a ReverTra Ace qPCR RT kit (Toyobo, Osaka, Japan), and RT-qPCR was performed using SYBR Green Realtime PCR Master Mix (Toyobo, Osaka, Japan) on a Roche LightCycler 480 II instrument (Roche, Basel, Switzerland). Relative mRNA quantification was performed using the ΔΔCt method, and *Tubb3* was used as an internal reference. Detailed primer sequences are listed in Table 1.

**TABLE 1:** Primers used for polymerase chain reaction (PCR).

Experiment	Gene	Primer sequence (5′ → 3′)
Mitochondrial DNA PCR (mtDNA PCR)	*Cox1*	GAGCAGGAATAGTAGGGA GTGTCTGATATTGGGTTAT
	*Hbb*	GGTGAACCCTGATGATGT TTTAGTGGTACTTGTGAGCC
RTq-PCR	*Nr4a1*	GCGGCTTTGGTGACTGGATAGAC AGTGATGAGGACCAGAGCAGACAG
	*Drp1*	ACAGCGTCCCAAAGGCAGTAATG CCATGTCCTCGGATTCAGTCAGAAG
	*Sirt1*	TAGGTTAGGTGGCGAGTA CAGCCTTGAAATCTGGGT
	*Sod2*	TCCCTGACCTGCCTTACGACTATG TCGTGGTACTTCTCCTCGGTGAC
	*Hmox1*	ACCTCCTCATTGTTATTGG TACTCGCCACCTAACCTA
	*Nrf2*	GCCTTCCTCTGCTGCCATTAGTC TGCCTTCAGTGTGCTTCTGGTTG
	*Tubb3*	CGTCCACCTTCATCGGCAACAG TCGGCCTCGGTGAACTCCATC

### Mitochondrial DNA Quantification

Total DNA was extracted using a DNeasy kit (Qiagen, Hilden, Germany). RT-qPCR was performed using SYBR Green Real-time PCR Master Mix (Toyobo, Osaka, Japan) on a Roche LightCycler 480 II instrument (Roche, Basel, Switzerland). The mitochondrial gene *Cox1* and nuclear gene *Hbb* were selected as representative genes of mitochondrial and nuclear DNA, respectively.

### Adenosine-Triphosphate Measurements

Fresh myocardial tissue samples (20 mg) were homogenized for tissue lysate extraction, and the tissue lysates were centrifuged at 12,000 × *g* for 5 min at 4°C. Myocardial tissue lysates were used to detect the ATP content (Beyotime, Shanghai, China) and protein concentrations. The obtained supernatant was mixed and incubated with the detection solution, and the ATP concentrations were measured using a luminometer. To normalize the results, the ATP levels were normalized to total protein concentrations (BCA assays).

### Intracellular Reactive Oxygen Species Assay

When H9c2 cells in 6-well plates reached 80% confluency, they were incubated with 2 mL total DMEM containing 10 μM DCFH-DA probes (Beyotime, Shanghai, China) for 1 h at 37°C. The harvested cells were kept on ice and immediately detected by flow cytometry. The FITC-fluorescent signal of 20,000 events was recorded using a BD FACSAria II instrument (BD, Oxford, United Kingdom). Data were analyzed using the FlowJo software.

### 10-*N*-Nonyl Acridine Orange Staining for Cardiolipin

To detect intracellular cardiolipin content, H9c2 cells were incubated with 100 μM NAO (a highly specific fluorescent probe for cardiolipin) (MedChemExpress, Shanghai, China) for 20 min at 37°C. After incubation, images were acquired using an Axio Scope A1 microscope (Zeiss, Oberkochen, Germany).

### Mitochondrial Membrane Potential Detection

To further assess the mitochondria of the H9c2 cells, mitochondrial membrane potential was determined with JC-1 staining. The H9c2 cells were seeded into 6-well plates and grown to 80% confluency, and then the cells were stained with the JC-1 staining kit (Beyotime, Shanghai, China) in accordance with the manufacturer’s instructions. The cell images were acquired using an Axio Scope A1 microscope (Zeiss, Oberkochen, Germany).

### Statistical Analysis

All data are shown as the mean ± standard deviation. A two-tailed Student’s *t*-test was used to compare variables between the two groups, and one-way or two-way analysis of variance was performed for multi-group comparisons. Statistical significance was set at *P* < 0.05. Statistical details are included in the respective figure legends.

## Results

### General Characteristics and Glucose Homeostasis in Rats With Obesity and Type 2 Diabetes Mellitus Was Significantly Ameliorated After Sleeve Gastrectomy

**FIGURE 1:**
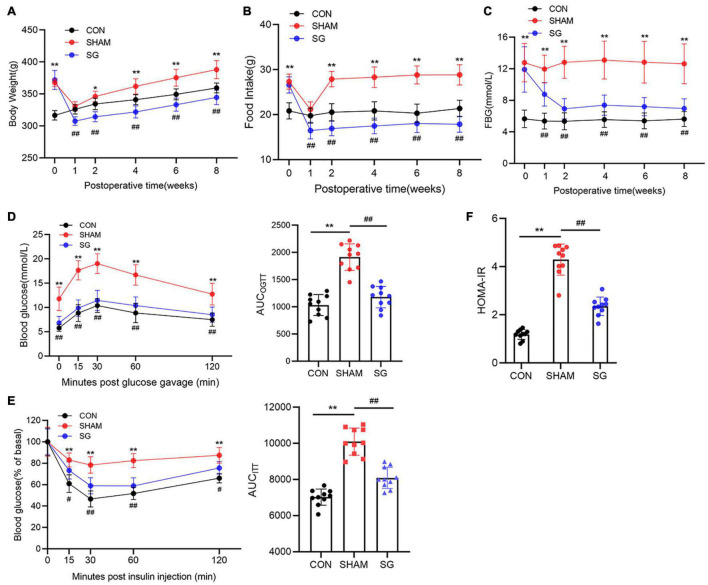
Metabolic alternations of sleeve gastrectomy (SG) on body weight, feeding behavior and glucose homeostasis in STZ-induced diabetic rats. **(A)** Body weight, **(B)** food intake, **(C)** and fasting blood glucose. **(D)** Curves of OGTT and **(E)** ITT were recorded 8 weeks post-operatively. AUC_*OGTT*_ and AUC_*ITT*_ was computed, respectively. **(F)** Values of HOMA-IR at 8 weeks after SG surgery. Data are presented as mean ± SD. *P < 0.05, **P < 0.01 CON vs. SHAM; ^#^*P* < 0.05, ^##^*P* < 0.01 SG vs. SHAM. *n* = 10 in each group.

As illustrated by the body weight curves, body weight in the sham surgery group and the SG group both bottomed out at 1 week after surgery and gradually increased thereafter. The sham and SG groups showed persistent differences (Figure 1A). Food intake after surgery was measured to determine changes in feeding behavior. The sham and SG groups consumed fewer calories during the first week after surgery, which may have resulted from the transient surgical stress. However, rats in the sham group were only hypophagic during the first week, while the appetitive deficit of rats in the SG group lasted for a long time, which enabled the maintenance of a reduced body weight (Figure 1B). To assess the effects of SG on glucose homeostasis, fasting blood glucose (FBG) concentrations were regularly recorded (Figure 1C). The FBG level diminished during the initial 14 days and remained stable thereafter. Rodents in the SG group displayed improvements in the capability to clear the oral gavage of glucose from blood, reflected as a 38% drop in the area under the curve (AUC) compared with sham-operated controls. An insulin tolerance test was performed, and it showed a lower AUC in SG rodents than in the SHAM group, demonstrating that SG further developed insulin sensitivity (Figures 1D,E). The HOMA-IR values fundamentally decreased after SG, which is consistent with the results above (Figure 1F).

### Reversion of Cardiac Dysfunction After Sleeve Gastrectomy

**FIGURE 2:**
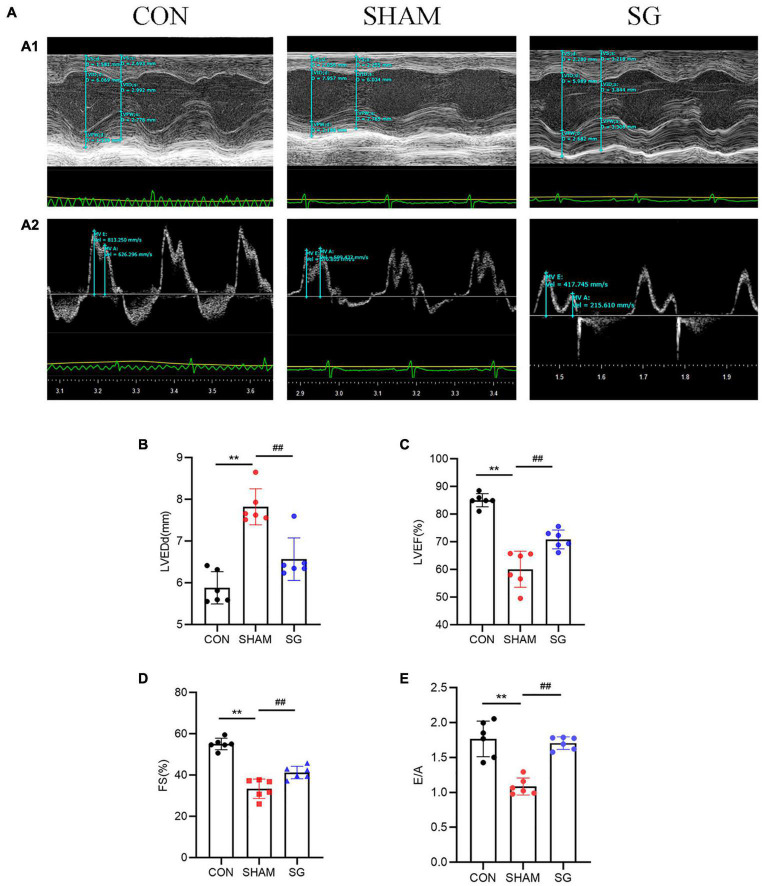
Effects of sleeve gastrectomy (SG) on cardiac function in rat models. **(A)** Echocardiography. **(A1)** Representative images of M-mode echocardiograms, **(A2)** Pulse-wave Doppler echocardiograms showed mitral inflow. Echocardiographic measurements including **(B)** LVEDd, **(C)** LVEF, **(D)** FS and **(E)** ratio of E/A. Data are presented as mean ± SD. **P < 0.01 CON vs. SHAM; ^##^*P* < 0.01 SG vs. SHAM. *n* = 6 in each group.

Echocardiographic assessment was done 2 months post-surgical procedure to survey heart function (Figure 2A). Rats that underwent sham surgery had a higher LVEDd, more prominent impaired LVEF, FS, and a higher E/A proportion compared to those in the control group. Echocardiographic examination revealed systolic and diastolic dysfunctions in the sham group. Nevertheless, in contrast to the sham group, it can be seen that SG surgery moderated these negative changes, as proven by the fundamentally diminished LVEDd with a reestablished LVEF, FS, and E/A proportion (Figures 2B–E).

### Myocardial Remodeling Was Reversed After Sleeve Gastrectomy

**FIGURE 3:**
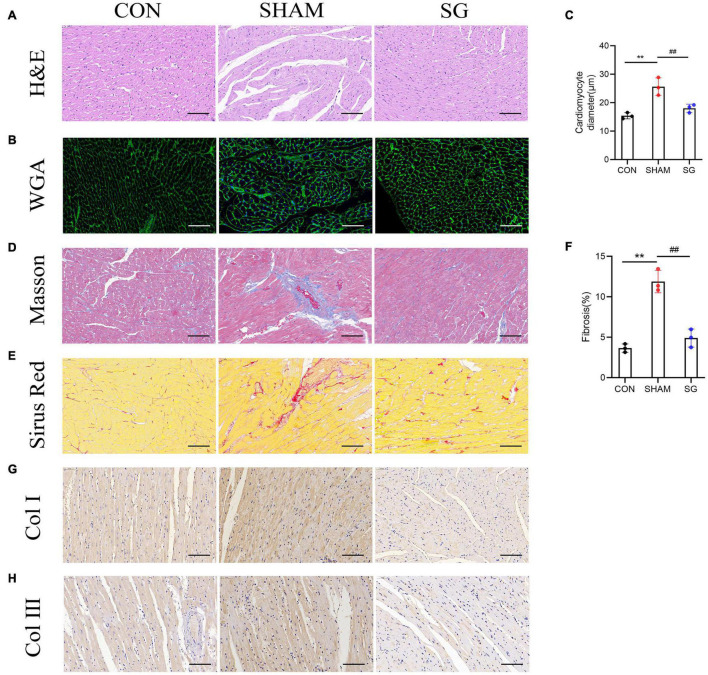
Effects of sleeve gastrectomy (SG) on diabetic-induced cardiac remodeling. **(A)** H&E staining images of left ventricle. **(B)** Plasma membrane stained with WGA. **(C)** Mean diameters of cardiomyocytes in each group **(D)** staining of masson trichrome, of which dark blue indicated collagen fibers. **(E)** Sirus red staining identified the dense collagen staining. **(F)** The percentage of fibrosis was calculated. Immunohistochemical staining of panel **(G)** collagen I, **(H)** collagen III. Scale bar, 50 μm. Data are presented as mean ± SD. **P < 0.01 CON vs. SHAM; ^##^*P* < 0.01 SG vs. SHAM. *n* = 3 in each group.

Diabetic cardiomyopathy is characterized by structural and functional derangement of myocardial tissue, comprising hypertrophy, myocardial fibrosis, and contractile dysfunction in both the diastolic and systolic phases. H&E staining, together with WGA-stained cardiomyocyte outlines, showed hypertrophy of cardiomyocytes in the sham group, which was attenuated after SG (Figures 3A–C). Sirius red and Masson’s trichrome staining revealed a rising level of cardiac fibrosis in the sham group, although SG surgery eased fibrosis (Figures 3D–F). In addition, IHC staining of collagen I and collagen III increased collagen deposition in the sham group, which was reversed after SG operation (Figures 3G,H). These results suggested that SG strikingly reversed DCM-induced myocardial remodeling.

### Sleeve Gastrectomy Ameliorated Oxidative Stress in the Cardiac Tissue of Diabetic Cardiomyopathy Rats

**FIGURE 4:**
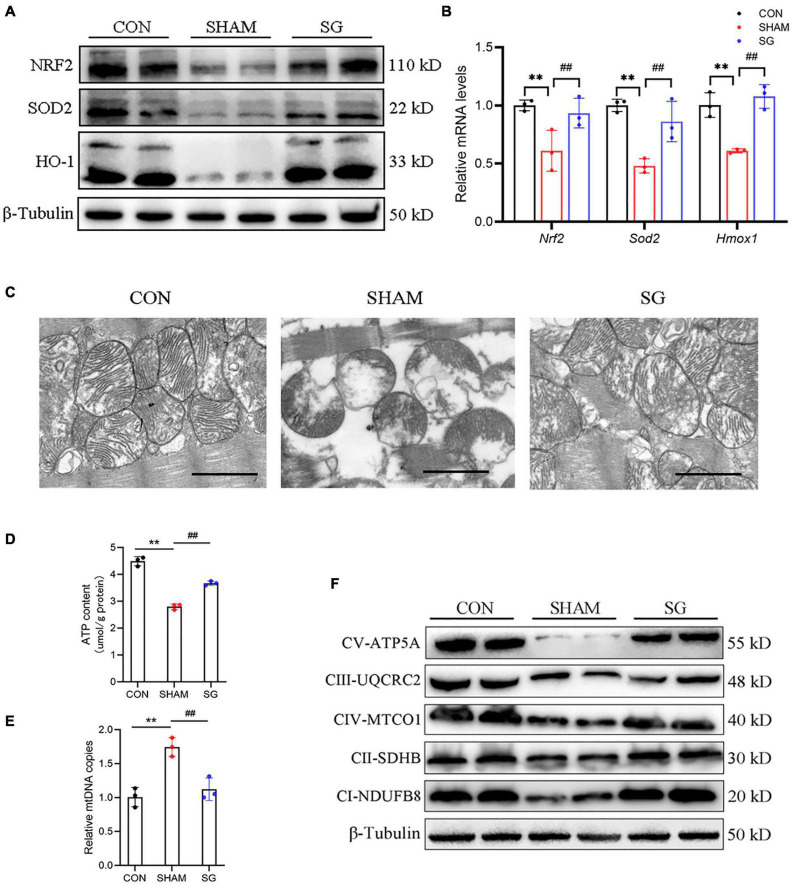
Sleeve gastrectomy (SG) attenuates oxidative stress and improves mitochondrial function in diabetic cardiomyopathy (DCM). **(A)** Protein levels of anti-oxidation molecules shown by western blot. β-Tubulin was an internal reference control. **(B)** Relative mRNA levels of anti-oxidation genes shown by real-time RT-PCR. *Tubb3* served as a reference gene. **(C)** Representative transmission electron micrographs. Scale bar, 1 μm. **(D)** ATP content in heart tissues. **(E)** Relative mtDNA copies per nuclear genome. **(F)** Protein levels of OXHPOS complexes in each group. Data are presented as mean ± SD. **P < 0.01 CON vs. SHAM; ^##^*P* < 0.01 SG vs. SHAM. *n* = 3 in each group.

It is well known that T2DM could increase the burden of oxidative stress (Civitarese et al., 2010). The rising generation of ROS and disabled clearance of ROS appear to contribute to the pathological progression of DCM (Huynh et al., 2014). Western blotting showed that multiple anti-oxidative proteins, including NRF2, SOD2, and HO-1, were suppressed in the sham rats and were upregulated after SG surgery (Figure 4A). The real-time PCR analysis yielded consistent results (Figure 4B). These data suggest that SG surgery could partially prevent oxidative stress in the myocardial tissues of rats with DCM.

### The Mitochondrial Function and Structure Were Improved With Sleeve Gastrectomy Performance

Reactive oxygen species are predominantly derived from the mitochondria in diabetes (Wang et al., 2017). We assessed mitochondrial structure and function. Transmission electron microscopy revealed disarranged sarcomeres, swollen mitochondria with disorganized cristae, and loss of intracellular substances in the heart of the sham group. In the SG group, layers of uniformly shaped mitochondria with copious and organized cristae intervened between regularly aligned myofibrils (Figure 4C). Heart tissues in the SG group showed higher ATP content than those in the sham-operated rats (Figure 4D). In addition, the copies of mtDNA in the myocardium were diminished with SG treatment compared to those in the sham group, indicating of a restoration of excessive mitochondrial fragmentation (Figure 4E). Consistently, we found that the proteins of respiratory complexes were upregulated after SG, indicating the restoration of oxidative phosphorylation (OXPHOS) (Figure 4F). Taken together, these results suggest that beneficial metabolic alterations may occur *via* the suppression of mitochondrial dysfunction after SG surgery.

### Sleeve Gastrectomy Activated Adenosine Monophosphate-Activated Protein Kinase Pathway and Nuclear Receptor Subfamily 4 Group A Member 1 Suppression for the Restoration of Mitochondrial Homeostasis

**FIGURE 5:**
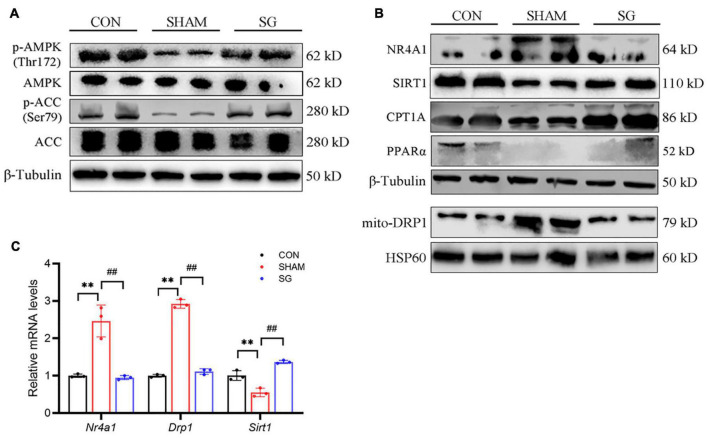
Sleeve gastrectomy (SG) activated adenosine monophosphate-activated protein kinase (AMPK) and depressed NR4A1/DRP1 expression for the restoration of mitochondrial homeostasis. **(A)** Protein levels of AMPK signaling shown by western blot. β-Tubulin was an internal reference control. **(B)** Western blot analysis of NR4A1/DRP1 signaling and CPT1α and PPARα. β-Tubulin was an internal reference control. **(C)** Relative mRNA levels of Nr4a1/Drp1 shown by real-time RT-PCR. *Tubb3* served as a reference gene. **P < 0.01 CON vs. SHAM; ^##^*P* < 0.01 SG vs. SHAM. *n* = 3 in each group.

Adenosine monophosphate-activated protein kinase has been proved to show a beneficial role of metabolic regulation in diabetes (Schaffer et al., 2015). Activation of the AMPK pathway suppresses NR4A1-mediated mitochondrial fission *via* DRP1 (Zhou et al., 2018; Wang et al., 2021). In this study, western blot showed that AMPK pathway was impaired in rats of DCM, as shown by decreased AMPK and ACC phosphorylation. Post-operatively, the AMPK pathway was activated in the heart tissues (Figure 5A). It has been reported that SIRT1, which can further downregulate NR4A1, can be activated by AMPK (Fulco et al., 2008; Qiang et al., 2011). Thus, we further determined the expression of SIRT1, NR4A1 and DRP1, and found that SIRT1 was overregulated after SG surgery, whereas the expression of NR4A1 and DRP1 in mitochondrial fractions was reduced post operation (Figure 5B). Similarly, real-time RT-PCR showed that the relative transcriptional levels of Sirt1, Nr4a1, and Drp1 were consistent with the protein levels (Figure 5C). Fatty acid oxidation in cardiomyocytes was enhanced after SG surgery, indicated by an increased expression of CPT-1α and PPARα (Figure 5B). Taken together, these results indicate that AMPK signaling activation and NR4A1 suppression may mediate the effect of SG on maintaining cardiac mitochondrial homeostasis.

### Adenosine Monophosphate-Activated Protein Kinase Mediated the Protective Effect of Mitochondrial Function in H9c2 Cells

To further testify the role of AMPK signaling in DCM models, we stimulated H9c2 cells with HG along with PA to mimic the hyperglycemic and hyperlipidemia environment. A-769662, an AMPK activator, was used to evaluate the effects of AMPK on diabetic cardiomyocytes.

**FIGURE 6:**
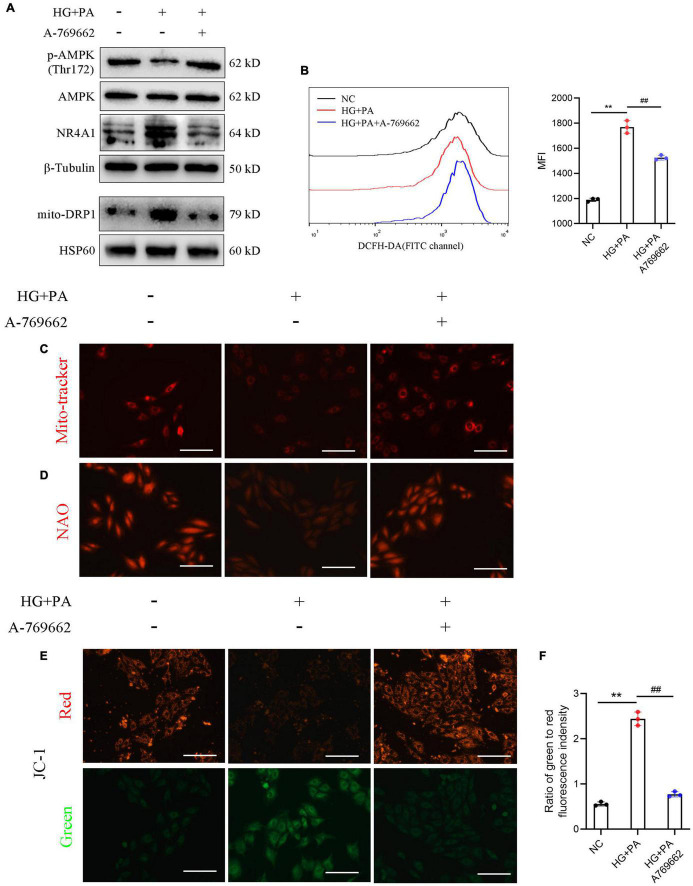
Adenosine monophosphate-activated protein kinase (AMPK) mediated the protective effect of mitochondrial function in H9c2 cells. **(A)** Protein levels of AMPK signaling. β-Tubulin was an internal reference control. **(B)** Reactive oxygen species (ROS) levels detected by flow cytometry with DCFH-DA probe. **(C)** Mito-tracker staining and **(D)** 10-*N*-nonyl acridine orange (NAO) staining **(E)** membrane potential shown by the ratio of intensity with JC-1 dye at 529 nm (green) to 590 nm (red). **P < 0.01 NC vs. HG + PA; ^##^*P* < 0.01 HG + PA vs. HG + PA + A-769662. *n* = 3 in each group.

Our data revealed that the protein levels of p-AMPK and SIRT1 were downregulated under HG + PA stimulation and were restored with an AMPK activator. The protein levels of NR4A1 and DRP1 were up-regulated in the HG + PA condition, whereas they were suppressed by AMPK activation (Figure 6A). We also determined the intracellular ROS levels with a redox-sensitive fluorescent dye, DCFH-DA, using flow cytometry. HG + PA markedly upregulated the fluorescent staining of ROS, whereas ROS levels were partially cleared following AMPK activation (Figure 6B). The Mito-tracker probe was used to stain the morphology of the mitochondria, and our data revealed that mitochondria were impaired with HG + PA stimulus, which could be ameliorated by an AMPK activator (Figure 6C). Cardiolipin (CL) is vital for oxidative phosphorylation because its oxidation is related to ROS accumulation and mitochondrial dysfunction (Li et al., 2010). Using NAO fluorescent staining, we found that oxidative damage to the CL was suppressed by AMPK activation (Figure 6D). Mitochondrial fission has been reported to induce depolarization of the mitochondrial membrane (Baixauli et al., 2011). Thus, we surveyed membrane potential using JC-1 fluorescent dye and found that AMPK protected cardiomyocytes from membrane potential disruption induced by HG + PA treatment (Figures 6E,F). These data suggest that AMPK preserves mitochondrial function in cardiomyocytes *in vitro*.

## Discussion

Diabetic cardiomyopathy is a pivotal cause of morbidity and mortality in patients with T2DM, and is defined by morphological, functional, and metabolic transformations in the heart as a significant cardiovascular complication in diabetic patients (Adeghate and Singh, 2014; Bugger and Abel, 2014). Ample evidence has showed that bariatric surgery had significant metabolic advantages in obesity, T2DM, and following complications (Ikramuddin et al., 2018; McGlone et al., 2020). In the present study, we induced DCM rat models by HFD feeding followed with low-dose intraperitoneal injection of STZ. We performed SG surgery or sham surgery in rats with DCM and compared the general metabolic characteristics to ensure that SG surgery had beneficial effects in the remission of T2DM and obesity. Additionally, as shown by echocardiography, SG partially restored cardiac function in rats with DCM. Histological assessment indicated reversion of myocardial remodeling and cardiac hypertrophy after SG surgery. We detected several indicators of oxidative stress in the myocardium, suggesting that SG surgery remarkably relieved the ROS burden in cardiac tissues. When emphasizing mitochondria, SG surgery imposed favorable effects on both the structure and function of the mitochondria. Further mechanistic *in vivo* and *in vitro* studies revealed that AMPK-mediated NR4A1 repression and subsequent mitochondrial improvement might be the underlying mechanism of the therapeutic effect of SG surgery on DCM.

In the present study, we have adopted the routine HFD feeding combined with low-dose STZ injection intraperitoneally to establish the DCM model. Because it mimics the regular pathological progression of T2DM, this technique has been widely utilized by numerous specialists (Reed et al., 2000; Sahin et al., 2007; Skovsø, 2014). In addition, this method is ordinarily adopted in the induction of DCM models (Ménard et al., 2010; Ti et al., 2011). In accordance with the findings of previous research (Chambers et al., 2011), metabolic improvement of SG procedure was observed in our study, shown by a decreased body weight, food intake, and FBG level and improved glucose tolerance and insulin sensitivity post-operatively. These data confirmed the impact of the SG operation (Figure 1).

Diabetic cardiomyopathy is initially characterized by diastolic dysfunction, which may precede systolic dysfunction (Brooks et al., 2008). Using cardiac echocardiography, cardiac function was identified in this study, and we found that diastolic and systolic function, which was impaired in DCM rats, improved after SG surgery. The pathogenesis of DCM remains disputable, and several mechanisms have been proposed, including myocardial hypertrophy, fibrotic remodeling, oxidative stress, and superfluous lipid deposition. Myocardial hypertrophy contributes to impaired contractility and reduced myocardial compliance (Aneja et al., 2008). In this study, SG surgery significantly alleviated cardiac hypertrophy, as shown by the reduced diameters of the cardiomyocytes. Interstitial and perivascular fibrosis in heart biopsies contribute to the impairment of contractility (Regan et al., 1977). Excessive collagen deposition in both the interstitial and perivascular regions was strikingly reduced post-operatively. These data reveal that owing to morphological improvements, functional benefits were obtained with SG (Figures 2, 3).

Mitochondrial dysfunction, abnormal mitochondrial ultrastructure, and the accumulation of oxidative stress have been reported to participate in the progression of DCM, characterized by impaired mitochondrial respiratory capacity and increased mitochondrial oxidative stress (Bugger and Abel, 2014). Under physiological conditions, mitochondrial fragmentation can increase ATP production to fulfill the energetic needs of the heart (Coronado et al., 2018). Nevertheless, superfluous mitochondrial fission causes disruption of the membrane potential, subsequently leading to mitochondrial dysfunction, cytochrome c leakage, and increased mitochondrial ROS accumulation (Veeranki et al., 2016; Ding et al., 2018). Our data confirmed that SG surgery could alleviate the abnormal mitochondrial ultrastructure, and could upregulate the impaired expression of anti-oxidative signaling proteins, which implied a clearance of overburdened ROS. In addition, our results showed that SG could decrease number of mtDNA copies, which might reveal the amelioration of excessive mitochondrial fragmentation or mitochondrial biogenesis and might be the key contributor to cardiac recovery. Also, ATP content was increased after SG performance, along with the fact that SG restored the expression of respiratory complexes in OXPHOS. That is, we determined that SG surgery suppressed pathologically excessive fragmentation in mitochondria and restored respiratory capability in DCM (Figure 4).

Previous studies have reported that SG can activate AMPK signaling in adipose and pancreatic beta cells to gain metabolic benefits (Liu et al., 2020; Li et al., 2021). SIRT1/AMPK activation ameliorated the development and progression of DCM (Packer, 2020b). In this study, we confirmed that SG promotes the activation of SIRT1/AMPK signaling. As has been shown in previous studies, AMPK can inhibit the expression of NR4A1, which seems as a culprit factor in the heart, as it promotes mitochondrial fragmentation and decreases the mitochondrial membrane potential to result in the disruption of mitochondrial homeostasis (Zhou et al., 2018; Wang et al., 2021). *In vivo* experiments showed that SG could decrease the expression of NR4A1 and DRP1, accompanied with enhanced fatty acid oxidation in cardiomyocytes. AMPK agonists suppress NR4A1 and DRP1 in *in vitro* models, along with a decrease in ROS levels and restoration of mitochondrial structure and homeostasis. Taken together, these data imply that the therapeutic effects of SG surgery on DCM may be mediated by restraining mitochondrial homeostasis. Moreover, *in vitro* findings suggested that AMPK/NR4A1 suppression-mediated mitochondrial homeostasis might be the underlying mechanism in the SG treatment of DCM (Figures 5, 6).

Although the pathological progression of DCM is initiated and exacerbated by hyperglycemia and insulin resistance, blood glucose control has only limited effects on the alleviation of cardiovascular diseases (Brown et al., 2010). Additionally, a clinical trial showed that hypoglycemic agents, such as GLP-1R agonists and DPP-4 inhibitors, could not reduce the risk of heart failure (Murtaza et al., 2019). Therefore, we have speculated that despite the hypoglycemic effects of SG surgery, the favorable metabolic effects of SG on DCM have some deep mechanisms. Activation of AMPK signaling and restoration of mitochondrial homeostasis might be vital mechanisms.

This study has several limitations. Firstly, these results were based on an obese diabetic rat model; the relevance of this animal model with obese patients with DCM undergoing bariatric surgery needs to be further investigated. Secondly, the two general effects of SG are the weight loss and improvements in glucose metabolism, which both contribute to the amelioration of DCM development. So, it is tough to define which factor plays the most direct role. Experiments will be required in the future to directly associate SG with cardiac performance *via* AMPK activation.

Taken together, ROS overburden and mitochondrial dysfunction ascribing to NR4A1/DRP1 induced mitochondrial fission and fragmentation play significant roles in the progression of DCM. SG surgery could activate AMPK and decrease NR4A1 levels, correct mitochondrial dysfunction, and enhance myocardial energy production to protect against myocardial remodeling and dysfunction. Whether this restoration of mitochondrial homeostasis and its upstream mechanisms in SG operation could be used as a new therapeutic target for DCM remains to be elucidated.

## Data Availability Statement

The original contributions presented in the study are included in the article/supplementary material, further inquiries can be directed to the corresponding author.

## Ethics Statement

The animal study was reviewed and approved by the Institutional Animal Care and Use Committee of Shandong Qianfoshan Hospital Affiliated to Shandong University.

## Author Contributions

SL, MZ, and GZ contributed to the conception of the study. SL, SD, QX, and BS performed the experiments. SL, LL, and WZ analyzed the data. SL and SD contributed to the drafting of the work. JZ and YC helped to perform the analysis with constructive discussions. All authors contributed to the article and approved the submitted version.

## Conflict of Interest

The authors declare that the research was conducted in the absence of any commercial or financial relationships that could be construed as a potential conflict of interest.

## Publisher’s Note

All claims expressed in this article are solely those of the authors and do not necessarily represent those of their affiliated organizations, or those of the publisher, the editors and the reviewers. Any product that may be evaluated in this article, or claim that may be made by its manufacturer, is not guaranteed or endorsed by the publisher.
